# New records and an updated list of reptiles from Thanh Hoa Province, Vietnam

**DOI:** 10.3897/BDJ.12.e134976

**Published:** 2024-11-15

**Authors:** Vinh Quang Dau, Thao Ngoc Hoang, Truong Quang Nguyen, Anh Van Pham

**Affiliations:** 1 Hong Duc University, Thanh Hoa, Vietnam Hong Duc University Thanh Hoa Vietnam; 2 Institute of Ecology and Biological Resources, Vietnam Academy of Science and Technology, 18 Hoang Quoc Viet Road, Hanoi, Vietnam Institute of Ecology and Biological Resources, Vietnam Academy of Science and Technology, 18 Hoang Quoc Viet Road Hanoi Vietnam; 3 Graduate University of Science and Technology, VAST, 18 Hoang Quoc Viet Road, Hanoi, Vietnam Graduate University of Science and Technology, VAST, 18 Hoang Quoc Viet Road Hanoi Vietnam; 4 Faculty of Environmental Sciences, University of Science, Vietnam National University, Hanoi, 334 Nguyen Trai Road, Thanh Hoa, Vietnam Faculty of Environmental Sciences, University of Science, Vietnam National University, Hanoi, 334 Nguyen Trai Road Thanh Hoa Vietnam

**Keywords:** distribution, Pu Luong Nature Reserve, morphology, reptiles, taxonomy, Xuan Lien Nature Reserve

## Abstract

**Background:**

Thanh Hoa Province is located in North Central Vietnam and the Province contains a large area of 393,361.33 hectares of natural forest. A complexity of landforms has given this Province a high level of biodiversity potential. However, the reptile fauna of Thanh Hoa Province is still underestimated. Previous studies documented 111 species of reptiles from this Province.

**New information:**

As a result of our field surveys in Pu Luong and Xuan Lien Nature Reserves, we report five species of reptiles for the first time from Thanh Hoa Province, Vietnam, namely *Plestiodontamdaoensis*, *Scincelladevorator* (Scincidae), *Elaphetaeniura*, *Pareasmacularius* and *Pseudoxenodonmacrops* (Colubridae), with novel data about morphological characteristics. In addition, we provide an updated checklist of 116 species of reptiles from Thanh Hoa Province. The reptile fauna of Thanh Hoa Province also contains a number of species of conservation concern: 22 species listed in the Red Data Book of Vietnam, 22 species listed in the IUCN Red List, 18 species listed in the Vietnam Governmental Decree No. 84; and 21 species listed in CITES appendices.

## Introduction

The reptile fauna is not only an important component of the global biodiversity, but also serves as important bioindicator for ecosystem health (Marsili et al. 2009). With a total of about 540 recognised species of reptiles, Vietnam is recognised as one of the most well-known countries in the world in terms of reptile diversity (Nguyen et al. 2009, Uetz et al. 2024). Since 2000, more than 100 new species have been described from the country (Nguyen et al. 2009, Uetz et al. 2024). Being part of the Indo-Burma biodiversity hotspot, northern Vietnam hosts many natural reserves and national parks. It shares a boundary with Laos and China. The tropical rainforest in northern Vietnam is biologically intriguing due to the high species endemism and richness. For example, about ten endemic reptile species have been reported from this area (Nguyen et al. 2015, Nguyen et al. 2017, Pham et al. 2019, Pham et al. 2023); however, as unprecedented numbers of new species are being discovered, reptiles are threatened by extinction (Rowley et al. 2010, Böhm M et al. 2013). Globally, IUCN (2024) estimated that about 20% of reptile species in the world are threatened with extinction and the principal threats to the reptile populations are habitat loss and degradation, followed by unsustainable wildlife trade, invasive species, pollution, diseases and climate change. In Vietnam, taxonomic, distribution and ecological information of many reptiles is still very limited, particularly in the remote areas like the north-western region and for species in inaccessible habitats, such as fossorial taxa or forest canopy specialists (Ziegler et al. 2008, Nguyen et al. 2010). Thanh Hoa Province is located in northern Vietnam with an area of 11,129.48 km^2^ (The People's Committee of Thanh Hoa Province 2015). The Province contains a large area of 393,361.33 hectares of natural forest with three natural reserves (Pu Hu, Pu Luong and Xuan Lien), Ben En National Park and Nam Dong Valuable Gymnosperm Conservation Area (The People's Committee of Thanh Hoa Province 2015). A complexity of landforms has given this Province a high level of biodiversity potential (The People's Committee of Thanh Hoa Province 2015). In terms of lizard and snake diversity, Thanh Hoa Province is poorly studied in Vietnam (Hoang et al. 2020). In their herpetofaunal list of Vietnam, Nguyen et al. (2009) recorded 23 species of lizards, 26 species of snakes and 11 species of turtles from this Province. Pham et al. (2012), Nguyen et al. (2015), Nguyen et al. (2018) and Luu (2022) documented eight new provincial records of lizards. Nguyen et al. (2011), Pham et al. (2012), Nguyen et al. (2015), Dau et al. (2019), Luu et al. (2019), Luu (2022), and Ha et al. (2024) documented 33 new provincial records of snakes. Nguyen et al. (2011), Pham et al. (2012), Nguyen et al. (2015) and Hoang et al. (2020) documented four new provincial records of turtles. In Thanh Hoa Province, a new gecko species was described from Pu Hu NR, viz. *Cyrtodactyluspuhuensis* Nguyen, Yang, Le, Nguyen, Orlov, Hoang, Nguyen, Jin, Rao, Hoang, Che, Murphy & Zhang (Nguyen et al. 2014).

In this study, our objectives are to: (1) report the new records of reptiles from Thanh Hoa Province, based on newly-collected materials from Pu Luong and Xuan Lien Nature Reserves, (2) Provide an updated checklist of species of reptiles from Thanh Hoa Province and (3) assess the conservation status of the reptiles from Thanh Hoa Province using the IUCN Red List criteria, Red Data Book of Vietnam, the Vietnam Governmental Decree No. 84 and CITES appendices.

## Materials and methods

**Sampling**

Field surveys were conducted in Xuan Lien Nature Reserve (NR) from 15 to 19 June 2007 by A.V Pham and in Pu Luong NR from 25 to 30 June 2023 by V.Q Dau and T.N Hoang and from 11 to 18 July 2023 by V.Q Dau. In addition, pitfall traps were also set up in Pu Luong NR from 3 November 2023 to 2 January 2024 and from 13 June to 13 July 2024 by V.Q Dau and T.N Hoang. The coordinates (WGS 84) and elevations were determined by using the GPS Garmin 62SX.

Field surveys were permitted by the Directorates of Pu Luong NR (permit No. 05/KBTPL issued on 27 July 2023). Specimens were collected by hand between 08:00 h and 22:00 h. Pitfall traps were checked every two days. Specimens were euthanised in a closed vessel with a piece of cotton wool containing ethyl acetate (Simmons 2002), fixed in 80% ethanol for five hours and then transferred to 70% ethanol for permanent storage. Voucher specimens were subsequently deposited in the collections of the Faculty of Nature Sciences, Hong Duc University (HDU), Thanh Hoa Province and Faculty of Environmental Sciences, University of Science (HUS), Vietnam National University, Hanoi (VNU).

For taxonomic identification, we referred to the descriptions of Smith (1935), Bourret (1937), Smith (1943), Taylor (1965), Hikida et al. (2001), Darevsky et al. (2004), Hecht et al. (2013), Pham et al. (2014), Pham et al. (2015), Nguyen et al. (2016), Pham et al. (2020), Luu (2022), and Ha et al. (2024). For species names, we followed Nguyen et al. (2009) and Uetz et al. (2024).

**Morphological characters**

Measurements were taken with a digital calliper to the nearest 0.1 mm. Terminology of morphological characters followed Nguyen et al. (2011) for skinks and Pham et al. (2020) for snakes: SVL: snout-vent length, from tip of snout to anterior margin of cloaca; TaL: tail length, from posterior margin of cloaca to tip of tail. Bilateral scale counts were given as left/right.

Statistic analyses were performed with the PAST Statistics software version 2.17 (Hammer et al. 2001). The Sorensen coefficient was used to compare the similarity of the species compositions between natural reserves (Pu Hu, Pu Luong and Xuan Lien), Ben En National Park and Nam Dong Valuable Gymnosperm Conservation Area. The lists of amphibian species from nearby protected areas were obtained from the literature, i.e. Nguyen et al. (2009), Nguyen et al. (2011), Pham et al. (2012), Nguyen et al. (2014), Nguyen et al. (2015), Nguyen et al. (2016), Dau et al. (2019), Luu et al. (2019), Hoang et al. (2020), Luu (2022), and Ha et al. (2024). This index is measured by the following formula: \begin{varwidth}{50in}\begin{equation*}
            djk = {2M \over 2M + N}
        \end{equation*}\end{varwidth}, in which M is the number of species occurring in both regions and N is the total number of species with presence in just one region.

## Taxon treatments

### Plestiodon tamdaoensis

(Bourret, 1937)

BDA68AD0-D1BF-51B7-A531-5A96E3AC36D4

#### Materials

- **Type status:** Other material. **Occurrence:** catalogNumber: JJLR01684; individualCount: 1; sex: juvenin; occurrenceID: CFDA4CA4-7F40-5DA9-90A9-F3144F82C801; **Taxon:** scientificNameID: *Plestiodontamdaoensis*; scientificName: *Plestiodontamdaoensis*; class: Reptilia; order: Squamata; family: Scincidae; genus: Plestiodon; specificEpithet: tamdaoensis; scientificNameAuthorship: (Bourret, 1937); **Location:** country: Vietnam; countryCode: VN; stateProvince: Thanh Hoa; county: Thanh Hoa; municipality: Ba Thuoc; locality: Near Thanh Lam Commune; verbatimElevation: 1385; verbatimLatitude: 20°27'25.95"N; verbatimLongitude: 105° 610.69"E; verbatimCoordinateSystem: WGS84; **Event:** eventDate: July; eventTime: 2023; eventRemarks: collected by V. Q. Dau; **Record Level:** language: en; collectionCode: Reptilia; basisOfRecord: PreservedSpecimen

#### Description

Morphological characters of the specimen from Thanh Hoa Province agreed well with the descriptions of Bourret (1937), Hikida et al. (2001) and Hecht et al. (2013): SVL 38.5 mm; TaL 38.6 mm (n = 1, subadult). Supranasals large, in contact with each other; postnasal single; postmentals 2; prefrontals in contact with each other; 3/3 loreals; lower eyelid scaly; 4/4 supraoculars; 8/8 supraciliaries; frontoparietals in contact with each other; interparietal larger than frontoparietals; parietals separated; nuchals in 3 pairs; 8/8 supralabials; 7/7 infralabials; tympanum deeply sunk, with 3/3 small lobules on the anterior edge; dorsal scales smooth; mid-body scales in 24 rows; paravertebral scales 43; 52 transverse rows of ventrals, smooth; precloacals two, enlarged; fingers and toes meeting when adpressed; subdigital lamellae under fourth finger 14/14 and 19/19 under fourth toe.

**Figure 1.**
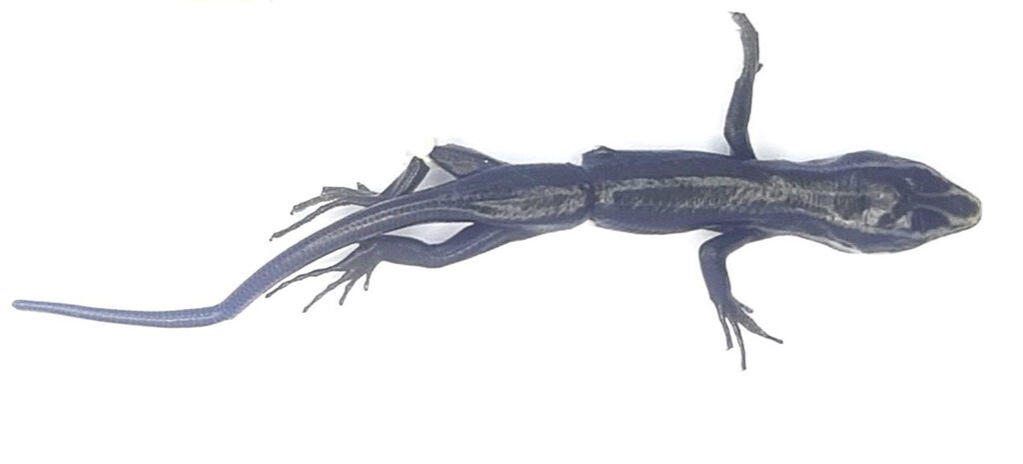
*Plestiodontamdaoensis* (subadult, HDU01684) from Thanh Hoa Province, Vietnam. Photo by A. V. Pham.

Colouration in preservative. Dorsal surface of head, body and tail brown with two cream stripes on head and three cream stripes on body; lateral band black-brown; ventral surface light brown (Fig. 1).

#### Distribution

In Vietnam, this species has been recorded from Ha Giang, Cao Bang, Bac Kan, Vinh Phuc, Bac Giang, Hai Duong, Son La, Hoa Binh and Nghe An Provinces (Nguyen et al. 2009, Uetz et al. 2024). Elsewhere, the species is known from China (Uetz et al. 2024).

Type locality: Tamdao, Vinh Phuc Province, Vietnam (Uetz et al. 2024).

#### Ecology

The specimen was found at 10:00 h on the ground. The surrounding habitat was bamboo forest and shrub.

### Scincella devorator

(Darevsky, Orlov & Cuc, 2004)

851E41FF-4E5A-56FA-8EA0-1F335C1F9A65

#### Materials

- **Type status:** Other material. **Occurrence:** catalogNumber: HUS.2024.11; individualCount: 1; sex: female; lifeStage: adult; occurrenceID: 4D9715EA-ACA8-55B3-AAC6-293AAB1B4C82; **Taxon:** scientificNameID: *Scincelladevorator*; scientificName: *Scincelladevorator*; genus: Scincella; specificEpithet: *devorator*; scientificNameAuthorship: (Darevsky, Orlov & Cuc, 2004); **Location:** country: Vietnam; countryCode: VN; stateProvince: Thanh Hoa; county: Thanh Hoa; municipality: Xuan Lien; locality: near Bat Mot Commune; verbatimElevation: 950m; verbatimLatitude: 20°02'19.8"N; verbatimLongitude: 104°59'16.8"E; verbatimCoordinateSystem: WGS84; **Event:** eventDate: June; eventTime: 2007; eventRemarks: collected by A.V Pham; **Record Level:** language: en; collectionCode: Lizard; basisOfRecord: PreservedSpecimen

#### Description

Morphological characters of the specimen from Thanh Hoa Province, Vietnam, agreed with the descriptions of Darevsky et al. (2004) and Pham et al. (2015): SVL 54.0 mm, TaL 71.0 mm (n = 1). Head longer than wide; rostral wider than high; supranasals absent; prefrontals separated from each other by frontal; parietals in contact posteriorly; nuchal scales in 3 pairs, enlarged; 2/2 loreals; 8/8 supraciliaries; 4/4 supraoculars, followed by one small postsupraocular; 1/1 primary temporal; 2/2 secondary temporals; lower eyelid with a large undivided opaque window; 7/7 supralabials, the fifth and sixth below the eye; ear opening without projecting lobules; tympanum deeply sunk; mental wider than long; 6/6 infralabials; postmental undivided; 28 rows of mid-body scales; scales of two vertebral rows on the neck widened; dorsal scales between lateral stripes in ½ + 6 + ½ rows, smooth, larger than lateral scales; paravertebral scales 66; 64 transverse rows of ventrals, smooth; precloacals two, enlarged; medial subcaudals widened; limbs short, pentadactyl; fingers and toes meeting when adpressed; subdigital lamellae under fourth finger 13/13 and 17/17 under fourth toe.

**Figure 2.**
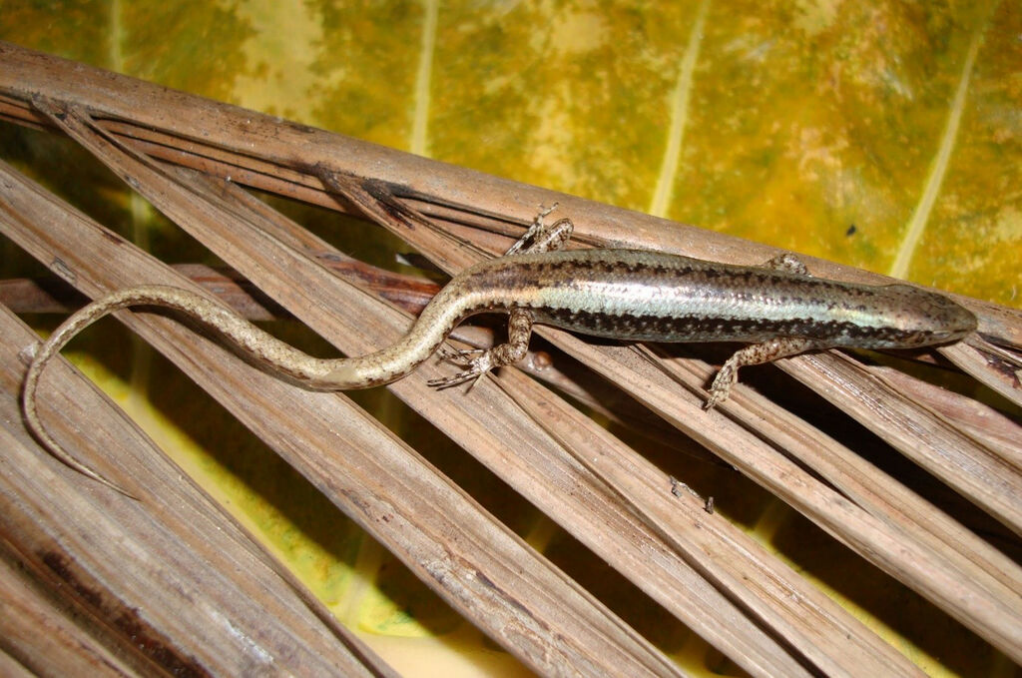
*Scincelladevorator* (adult female, HUS.2024.11) from Thanh Hoa Province, Vietnam. Photo by A. V. Pham

Colouration in preservative. Dorsal surface of head, body and tail base brownish-grey, with a dark vertebral stripe and two silver grey clear bands extending from parietals to base of tail; a distinct dark stripe from behind the eye to hind limb in upper lateral zone; venter and under surface of tail base cream (Fig. 2).

#### Distribution

In Vietnam, this species has been recorded from Quang Ninh, Bac Giang, Vinh Phuc and Son La Provinces (Darevsky et al. 2004, Pham et al. 2015, Ziegler et al. 2015). It should be stated that this species is endemic to Vietnam.

Type locality: Uong Bi, Quang Ninh Province (Uetz et al. 2024).

#### Ecology

The specimen was found at 16:00 h, under a carpet of fallen leaves. The surrounding habitat was disturbed evergreen forest of medium hardwood and shrub.

### Elaphe taeniura

Cope, 1861

64863227-891A-566B-96D6-D220AE9D2E1E

#### Materials

- **Type status:** Other material. **Occurrence:** catalogNumber: HDU6056; individualCount: 1; sex: female; lifeStage: adult; occurrenceID: 053D4A76-8439-514A-9AD2-78CA776C6943; **Taxon:** scientificNameID: *Elaphetaeniura*; scientificName: *Elaphetaeniura*; class: Reptilia; order: Squamata; family: Colubridae; genus: Elaphe; specificEpithet: *taeniura*; scientificNameAuthorship: Cope, 1861; **Location:** country: Vietnam; countryCode: VN; stateProvince: Thanh Hoa; county: Thanh Hoa; municipality: Ba Thuoc; locality: Near Thanh Son Commune; verbatimElevation: 1006m; verbatimLatitude: 20°48335’N; verbatimLongitude: 105°09964'E; verbatimCoordinateSystem: WGS84; **Event:** eventDate: Ortober; eventTime: 2024; eventRemarks: collected by V. Q. Dau and T.N. Thao; **Record Level:** language: en; collectionCode: Reptilia; basisOfRecord: PreservedSpecimen

#### Description

Morphological characters of the specimen from Thanh Hoa Province, Vietnam, agreed with the descriptions of Smith (1943) and Schulz (2010): SVL 506 mm, TaL 127 mm (n = 1). Body cylindrical; head moderately distinct from neck; eye large, pupil round; rostral as broad as high, visible from above; internasals broad larger than long; prefrontal about two-thirds of frontal; parietals as long as wide; nasal divided; loreal 1/1; preoculars 2/2; postoculars 3/3, bordering anterior temporals; anterior temporals 1/1; posterior temporals 2/2; supralabials 9/9, fifth and sixth touching the eye, eighth-largest; infralabials 12/12, first to sixth bordering chin shields; dorsal scale rows 25–25–21, keeled from the eighth row, laterally smooth; ventrals 275; cloacal scale divided; subcaudals 112, paired.

**Figure 3.**
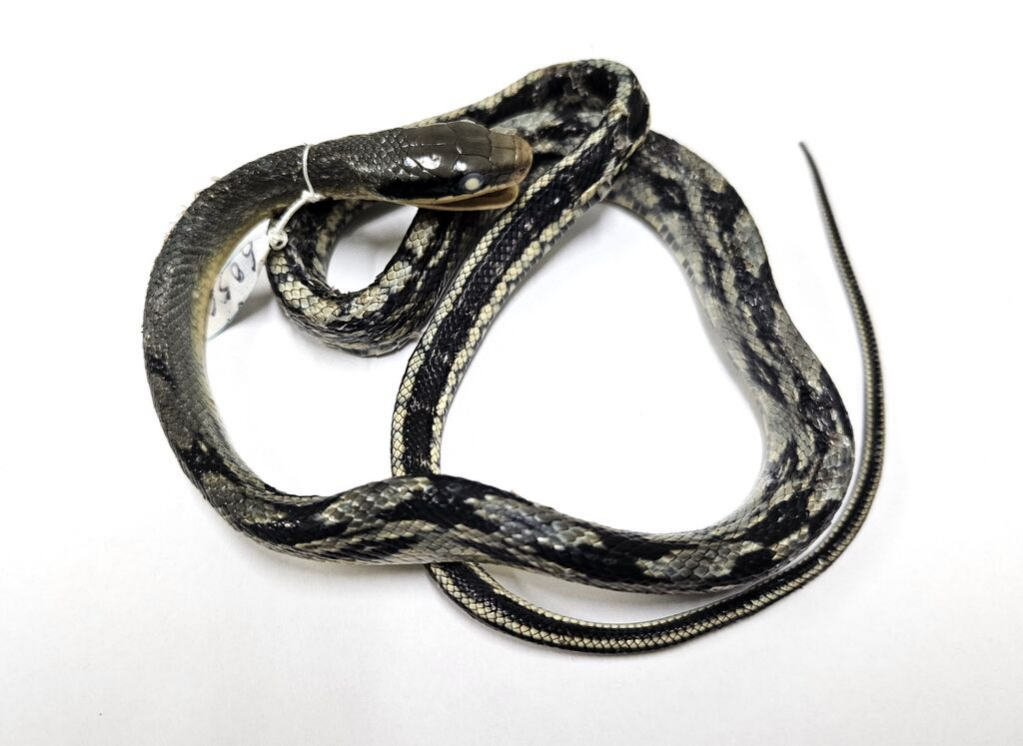
*Elaphetaeniura* (subadult, HDU6056) from Thanh Hoa Province, Vietnam. Photo by A. V. Pham.

Colouration in preservative. Dorsal surface of head and body brownish above, the head and neck uniform, with a black stripe on each side of the head, broadest behind the eye; anterior part of the back with a vertebral series of large black butterfly-shaped spots and smaller diamond-shaped ones on lateral; posterior part of back with a pale grey vertebral stripe, 3 or 4 scales wide and a broad black stripe on each side, 6 scales wide; this interrupted by light spots or transverse bars as far as the vent; ventral yellowish, with black spots in the outer margins. Tail black with four light stripes extending along each of the lateral, vertebral and ventral (Fig. 3).

#### Distribution

In Vietnam, this is a widespread species known, from Lao Cai and Cao Bang Provinces in the north, southwards to Kon Tum and Gia Lai Provinces (Nguyen et al. 2009). This species is also known from India, Bhutan, Burma, Thailand, Laos, Cambodia, Korea, Myanmar, W Malaysia, China, Taiwan, Indonesia and Japan (Uetz et al. 2024).

Type locality: China: Zhejiang, Ningbo; Ningpo and Siam; Siam, Ningpo (Uetz et al. 2024).

#### Ecology

The subadult was found at 20:00 h on the forest floor. The surrounding habitat was evegreen forest.

### Pareas macularius

Theobald, 1868

A6135692-192C-5454-831A-CDFC99E48BC0

#### Materials

- **Type status:** Other material. **Occurrence:** catalogNumber: JJLR01649; individualCount: 1; sex: female; lifeStage: adult; occurrenceID: BFAD93DA-CC3D-5688-9D52-74B1FFB933D4; **Taxon:** scientificNameID: *Pareasmacularius*; scientificName: *Pareasmacularius*; class: Reptilia; order: Squamata; family: Colubridae; genus: Pareas; specificEpithet: macularius; scientificNameAuthorship: Theobald, 1868; **Location:** country: Vietnam; countryCode: VN; stateProvince: Thanh Hoa; county: Thanh Hoa; municipality: Ba Thuoc; locality: Near Thanh Son Commune; verbatimElevation: 668m; verbatimLatitude: 20°29'7.37"N; verbatimLongitude: 105° 6'42.21"E; verbatimCoordinateSystem: WGS84; **Event:** eventDate: June; eventTime: 2023; eventRemarks: collected by V. Q. Dau and T.N. Thao; **Record Level:** language: en; collectionCode: Reptilia; basisOfRecord: PreservedSpecimen

#### Description

Morphological characters of the specimen from Thanh Hoa Province agreed well with the descriptions of Smith (1943) and Hauser (2017): SVL 385.0 mm, TaL 65.0 mm (n = 1). Body strongly compressed; head distinct from neck; nasal single; loreal 1/1; preocular 1/1; postocular 1/1; subocular 1, long and slender; anterior temporals 2/2; posterior temporals 3/3; supralabials 7/7, third to fifth below the eye, seventh very long; infralabials 7/7; mental groove absent; dorsal scale rows 15–15–15, with 11 median rows of keeled at mid-body keeled; ventrals 152; cloacal undivided; subcaudals 40, divided.

**Figure 4.**
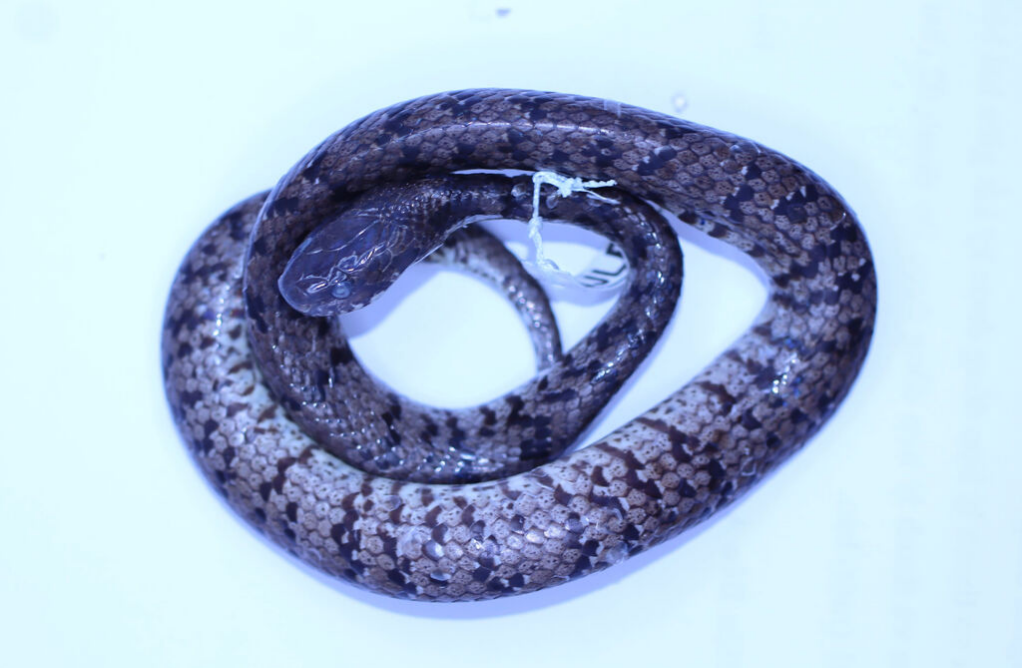
*Pareasmacularius* (adult male, HDU01649) from Thanh Hoa Province, Vietnam. Dorsal views. Photo by A. V. Pham

**Figure 5.**
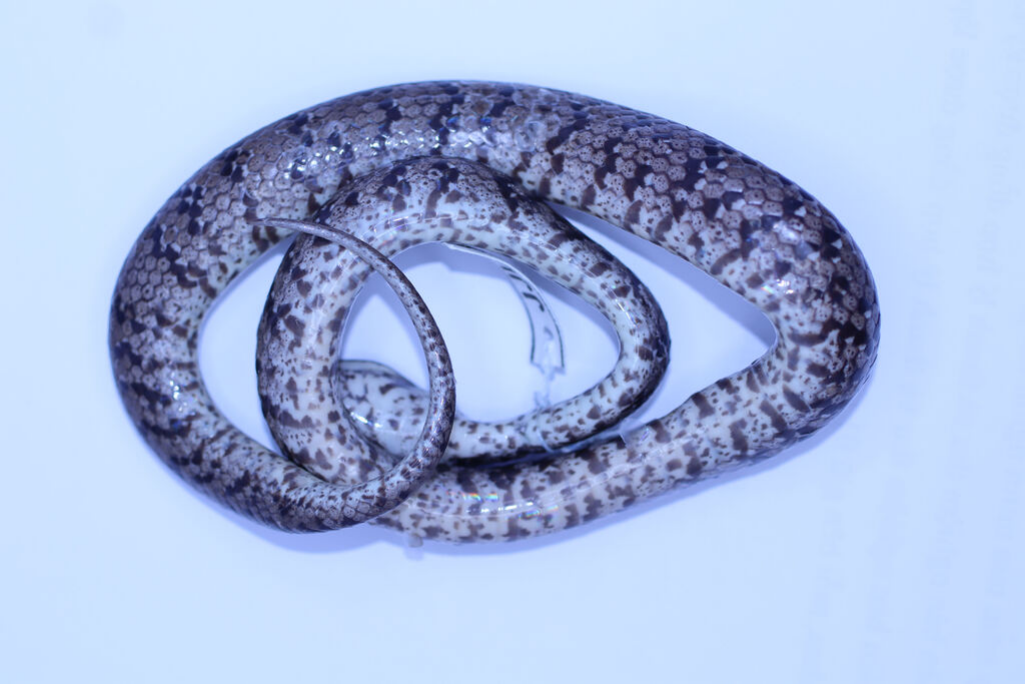
*Pareasmacularius* (adult male, HDU01649, belly side) from Thanh Hoa Province, Vietnam. Ventral views. Photo by A. V. Pham.

Colouration in preservative. Dorsal surface of head, body and tail grey-brown dorsally, with transverse bars on the back composed of black spots and small white dots; rudimentary brown on nuchal collar present; ventral light brown with many dark blotches (Figs 4, 5).

#### Distribution

In Vietnam, this species has been reported from Bac Kan, Cao Bang, Vinh Phuc, Hai Duong, Hoa Binh, Lai Chau, Nghe An and Quang Binh Provinces (Uetz et al. 2024). Elsewhere, the species has been reported from India, Myanmar, Thailand and China (Uetz et al. 2024).

Type locality: “Tenasserim” [= Tanintharyi Div., S Myanmar] (Uetz et al. 2024).

#### Ecology

The specimen was found at 20:00 h on the ground. The surrounding habitat was karst forest composed of small hardwoods, liane and shrub.

### Pseudoxenodon macrops

(Blyth, 1855)

DDD1DD54-1B48-56E0-BB0A-F4097DE25AD5

#### Materials

- **Type status:** Other material. **Occurrence:** catalogNumber: HDU5001; individualCount: 1; sex: Subadult; occurrenceID: EC18872D-893F-5981-B002-FF64F40C1956; **Taxon:** scientificNameID: Pseudoxenodonmacrops; scientificName: Pseudoxenodonmacrops; class: Reptilia; order: Squamata; family: Colubridae; genus: Pseudoxenodon; specificEpithet: macrops; scientificNameAuthorship: (Blyth, 1855); **Location:** country: Vietnam; countryCode: VN; stateProvince: Thanh Hoa; county: Thanh Hoa; municipality: Ba Thuoc; locality: Near Thanh Son Commune; verbatimElevation: 1006m; verbatimLatitude: 20°48335’N; verbatimLongitude: 105°09964'E; verbatimCoordinateSystem: WGS84; **Event:** eventDate: June; eventTime: 2024; eventRemarks: collected by V. Q. Dau; **Record Level:** language: en; collectionCode: Reptilia; basisOfRecord: PreservedSpecimen
- **Type status:** Other material. **Occurrence:** catalogNumber: HDU5002; individualCount: 1; sex: Subadult; occurrenceID: 3B88177D-B7BA-5718-B556-DF1F71DF3055; **Taxon:** scientificNameID: Pseudoxenodonmacrops; scientificName: Pseudoxenodonmacrops; class: Reptilia; order: Squamata; family: Colubridae; genus: Pseudoxenodon; specificEpithet: macrops; scientificNameAuthorship: (Blyth, 1855); **Location:** country: Vietnam; countryCode: VN; stateProvince: Thanh Hoa; county: Thanh Hoa; municipality: Ba Thuoc; locality: Near Thanh Son Commune; verbatimElevation: 1006m; verbatimLatitude: 20°48335’N; verbatimLongitude: 105°09964'E; verbatimCoordinateSystem: WGS84; **Event:** eventDate: July; eventTime: 2024; eventRemarks: collected by V. Q. Dau; **Record Level:** language: en; collectionCode: Reptilia; basisOfRecord: PreservedSpecimen

#### Description

Morphological characters of the specimens from Thanh Hoa Province agreed well with the descriptions of Smith (1943) and Pham et al. (2014): SVL 200.0–200.6 mm, TaL 37.0–40.0 mm (n = 2, subadults). Body cylindrical; head distinct from neck; snout elongate; rostral as broad as high, partly visible from above; internasals as wide as long, not in contact with loreal; prefrontal about half the length of frontal; frontal pentagonal; parietals longer than wide; nasal paired; loreal 1/1; supralabials 8/8, fourth to fifth entering orbit, seventh largest; infralabials 10/10, first to fourth bordering chin shields; preoculars 2/2; postoculars 2/2, bodering anterior temporals; anterior temporals 2/2; posterior temporals 2/2; dorsal scale rows 19–17–15, keeled; ventrals 158–160 in juvelines; cloacal scale paired; subcaudals 59–60, paired.

**Figure 6.**
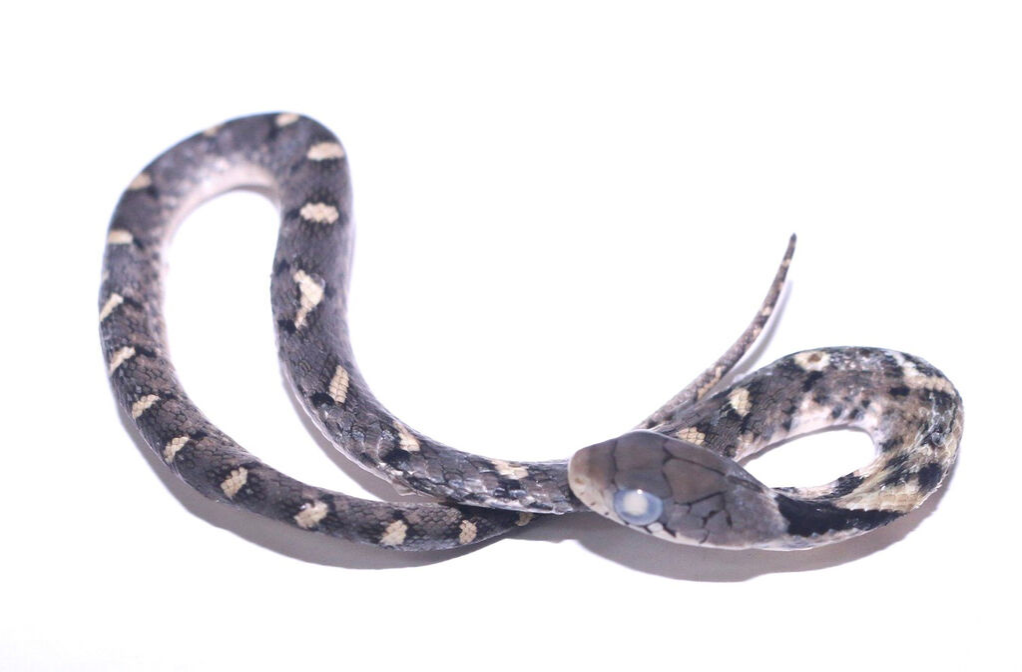
*Pseudoxenodonmacrops* (subadult, HDU51001) from Thanh Hoa Province, Vietnam. Photo by V. Q. Dau.

Colouration in preservative. Dorsal surface brownish-grey with short white cross-bars of black edge; a large V-shaped black spots on neck; chin and venter yellowish-cream, with large black spots anteriorly (Fig. 6).

#### Distribution

In Vietnam, this species has been recorded from Lai Chau, Lao Cai, Son La, Vinh Phuc, Nghe An, Ha Tinh, Quang Binh, Da Nang and Lam Dong Provinces (Pham et al. 2014). Elsewhere, this species is known from India, Nepal, China, Myanmar, Malaysia, Thailand and Laos (Nguyen et al. 2009, Uetz et al. 2024).

Type locality: "near Darjiling" [Darjeeling, E India] (Uetz et al. 2024).

#### Ecology

Two subadults were found between 09:00 h and 12:00 h on the ground, near a slow flowing stream. The surrounding habitat was evergreen secondary forest.

## Analysis

Statistical analyses showed that the species composition of the reptile fauna of Pu Luong NR is most similar to that of Ben En National Park (djk = 0.55319) with 21 species recorded in both areas, whereas it is only 0.42424 between Pu Luong NP and Xuan Lien NR (see Table 1).

## Discussion

Our new findings bring the total number of reptile species from Xuan Lien NR to 54 and that of Pu Luong NR to 37. The new records also bring the total number of reptiles species to 116 in Thanh Hoa Province, comprising 8 species of Agamidae, 8 species of Gekkonidae, 14 species of Scincidae, 2 species of Lacertidae, 1 species of Varanidae, 1 species of Pythonidae, 2 species of Typhlopidae, 1 species of Xenopeltidae, 51 species of Colubridae, 1 species of Pseudaspididae, 6 species of Elapidae, 2 Homalopsidae, 4 species of Viperidae, 1 species of Platysternidae, 2 species of Testudinidae, 9 species of Geoemydidae and 3 species of Trionychidae (Table 2). Nguyen et al. (2011) reported 36 reptiles species from Pu Hu NR, Thanh Hoa Province, including *Acanthosauracapra* Günther, *Cyrtodactylusirregularis* Smith, *Bronchocelavietnamensis* Hallermann & Orlov, *Varanidaenebulosus* Gray, *Ahaetullanasuta* Lacepède, *Pythonrecutilatus* Schneider, *Boigadendrophyla* Boie, *B.kraepelini* Stejneger, *Bungaruscandidus* Linaeus and *Calliophiskelloggi* Pope from adjacent areas. However, *C.irregularis* belongs to the species complex and subsequently described as *C.puhuensis* (Nguyen et al. 2014). *A.capra*, *B.vietnamensis*, *V.nebulosus*, *B.candidus* and *B.dendrophila* are only recorded in the south of Vietnam (Nguyen et al. 2009, Uetz et al. 2024). *Ahaetullanasuta* has not been recorded in Vietnam and only found in Sri Lanka (Uetz et al. 2024). Luu et al. (2019) described *Lycodonnamdongensis* in Nam Dong Valuable Gymnosperm Conservation Area; however, this species was considered as a junior synonym of *L.chapaensis* (Angel & Bourret) (Wang et al. 2020). *Pareasmacularius* was synonymised with *P.margaritophorus* (Huang 2004). However, genetic data suggested that it is a valid species (Zaher et al. 2019, Suntrarachun et al. 2020, Wang et al. 2020). *P.macularius* can be distinguished from *P.margaritophorus* by having keeled-scales on the back (vs. smooth in the latter) (Hauser 2017).

In terms of conservation concern, amongst 116 species of reptiles recorded in Thanh Hoa Province, 22 species are listed in the Red Data Book of Vietnam (Dang et al. 2007), with four species categorised as CR, eight species categorised as EN and 10 species categorised as VU; 22 species are listed in the IUCN Red List (IUCN 2024), with eight species categorised as CR, five species categorised as EN, seven species categorised as VU and two species categorised as NT; 18 species are listed in the Vietnam Governmental Decree No. 84/2021/ND-CP (The Government of Vietnam 2021), with five species included in the Group IB and 13 species in the Group IIB; and 21 species listed in CITES appendices (CITES 2023), with four species included in Appendix I and 18 species in Appendix II (Table 2). In addition, according to our observations in this study, major threats to the habitat and populations of reptiles in the Thanh Hoa Province are deforestation resulting from agricultural activities, illegal timber logging, free grazing of cattle in the forest and wildlife poaching for food and trade.

## Supplementary Material

XML Treatment for Plestiodon tamdaoensis

XML Treatment for Scincella devorator

XML Treatment for Elaphe taeniura

XML Treatment for Pareas macularius

XML Treatment for Pseudoxenodon macrops

## Figures and Tables

**Table 1.** Similarity (Sorensen coefficient index) of the species composition of the reptiles fauna between natural reserves (Pu Hu, Pu Luong and Xuan Lien), Ben En National Park and Nam Dong Valuable Gymnosperm Conservation Area.

	Ben En National Park	Xuan Lien NR	Pu Hu NR	Nam Dong, Valuable Gymnosperm Conservation Area	Pu Luong NR
Ben En National Park	1				
Xuan Lien NR	0.50485	1			
Pu Hu NR	0.69048	0.44944	1		
Nam Dong Valuable Gymnosperm Conservation Area	0.55652	0.56667	0.51485	1	
Pu Luong NR	0.55319	0.42424	0.45000	0.48649	1

**Table 2.** List of reptile species recorded from Thanh Hoa Province, Vietnam. * = new record for Thanh Hoa Province; 1 = Nguyen et al. (2009), 2 = Nguyen et al. (2011), 3 = Pham et al. (2012), 4 = Nguyen et al. (2014), 5 = Nguyen et al. (2015), 6 = Nguyen et al. (2016), 7 = Nguyen et al. (2018), 8 = Dau et al. (2019) (2019), 9 = Luu et al. (2019), 10 = Hoang et al. (2020), 11 = Luu (2022), 12 = Ha et al. (2024), 13 = This study. Decree 84 (2021) = The Governmental Decree No 84/2021/ND-CP, dated on 22 September 2021 by the Government of Vietnam on the management of endangered wild flora and fauna. Group IB: prohibited exploitation and use for commercial purpose and Group IIB: limited exploitation and use for commercial purpose (The Government of Vietnam 2021); RBVN (2007) = Vietnam Red Data Book CR = Critically Endangered, EN = Endangered, VU = Vulnerable (Dang et al. (2007)); IUCN (2024) = The IUCN Red List of Threatened Species. CR = Critically Endangered, EN = Endangered, VU = Vulnerable, LR/NT = Lower Risk/Near Threatened (IUCN 2024). CITES (2023) = Convention on international trade in endangered species of wild fauna and flora, Appendices I, II and III (CITES 2023).

**Name**	**Previous record**	**RBVN 2007**	**IUCN 2024**	**Decree 84/2021**	**CITES 2023**
SQUAMATA					
Agamidae					
*Acanthosauralepidogaster* (Cuvier, 1829)	1, 2, 3, 5, 10, 11				
*Calotesemma* Gray, 1845	1, 2, 5, 10, 11				
*Calotesmystaceus* Duméril & Bibron, 1837	5				
*Calotesversicolor* (Daudin, 1820)	1, 5, 10				
*Dracomaculatus* (Gray, 1845)	1, 2, 10, 11				
*Leiolepisbelliana* (Hardwicke & Gray, 1827)	1, 10				
*Leiolepisreevesii* (Gray, 1831)	1, 10	VU			
*Physignathuscocincinus* Cuvier, 1829	1, 2, 3, 5, 10, 11	VU	VU		II
Gekkonidae					
*Hemidactylusfrenatus* Duméril & Bibron, 1836	1, 2, 5, 10, 11				
*Hemidactylusgarnotii* Dumeril & Bibron,1836	11				
*Hemidactylusvietnamensis* Daversky, Kupriyanova & Kupriyanova, 1984	1, 2, 5, 10				
*Gehyramutilata* (Wiegmann, 1834)	2, 10				
*Gekkopalmatus* Boulenger, 1907	3, 5, 11				
*Gekkoreevesii* (Gray, 1831)	1, 2, 3, 5, 10, 11	VU			
*Cyrtodactylusbobrovi* Nguyen, Le, Pham, Ngo, Hoang, Pham & Zeigler, 2015	7				
*Cyrtodactyluspuhuensis* Nguyen, Yang, Thi Le, Nguyen, Orlov, Hoang, Nguyen, Jin, Rao, Hoang, Che, Murphy & Zhang, 2014	4, 11				
Scincidae					
*Eutropischapaensis* (Bourret, 1937)	1, 10				
*Eutropislongicaudatus* (Hallowell, 1857)	1, 2, 5, 10				
*Eutropismacularius* (Blyth, 1853)	1, 2, 5, 10, 11				
*Eutropismultifasciatus* (Kuhl, 1820)	1, 2, 5, 10, 11				
*Lygosomasiamense* Siler, Heitz, Davis, Freitas, Aowphol, Termprayoon & Grismer, 2018	1, 10				
*Plestiodonelegans* (Boulenger, 1887)	1, 10				
*Plestiodonquadrilineatus* Blyth, 1853	1, 5, 10				
*Plestiodontamdaoensis* (Bourret, 1937)*	13				
*Scincelladevorator* (Darevsky, Orlov & Cuc, 2004)*	13			
*Scincellareevesii* (Gray, 1838)	1, 10, 11				
*Sphenomorphuscryptotis* Darewsky, Orlov & Ho, 2004	3, 11				
*Sphenomorphusindicus* (Blyth, 1853)	1, 10, 11				
*Sphenomorphusmaculatus* (Blyth, 1853)	3				
*Tropidophorushainanus* Smith, 1923	3, 11				
Lacertidae					
*Takydromuskuehnei* Van Denburgh, 1909	1, 5, 10				
*Takydromussexlineatus* Daudin, 1802	1, 3, 5, 10, 11				
Varanidae					
*Varanussalvator* Laurenti, 1786	1, 2, 3, 10, 11	EN		IIB	II
Pythonidae					
*Pythonbivittatus* Kuhl, 1820	1, 2, 3, 6, 10, 12	CR		IIB	I
Xenopeltidae					
*Xenopeltisunicolor* Reinwartd, in Boie, 1827	1, 2, 6, 10, 12				
Typhlopidae					
*Indotyphlopsbraminus* (Daudin, 1803)	1, 2, 10, 11, 12				
*Argyrophisdiardii* (Schlegel, 1839)	1, 10				
Colubridae					
*Ahaetullaprasina* (Boie, 1827)	1, 2, 3, 5, 6, 10, 11, 12				
*Amphiesmastolatum* (Linnaeus, 1758)	1, 6, 10				
*Boigabourreti* Tillack, Ziegler & Khac Quyet, 2004	11, 12		EN		
*Boigaguangxiensis* Wen, 1998	6, 11, 12				
*Boigamultomaculata* (Boie, 1827)	2, 3, 5, 6, 11, 12				
*Calamariapavimentata* Duméril, Bibron&Duméril, 1854	5				
*Calamariaseptentrionalis* Boulenger, 1890	1, 3, 6, 10, 11, 12				
*Coelognathusradiatus* (Boie, 1827)	1, 2, 3, 6, 8, 10, 12	VU			
*Dendrelaphisngansonensis* (Bourret, 1935)	1, 10, 11, 12				
*Dendrelaphispictus* (Gmelin, 1789)	1, 6, 11, 12				
*Elaphemoellendorffi* (Boettger, 1864)	5	VU	VU		
*Elaphetaeniura* Cope, 1861	13		VU		
*Fowleaflavipunctatus* (Hallowell, 1860)	1, 2, 5, 6, 10, 12				
*Gonyosomaboulengeri* (Mocquard, 1897)	6, 12				
*Gonyosomacoeruleum* Liu, Hou, Lwin, Wang & Rao, 2021	11, 12				
*Gonyosomahainanense* (Peng, Zhang, Huang, Burbrink, Chen, Hou, Zhu, Yang, Wang, 2021)	11				
*Hebiusannamensis* Bourret, 1934	6			
*Hebiusboulengeri* (Gressitt, 1937)	6				
*Hebiuskhasiensis* (Boulenger, 1890)	11, 12				
*Liopeltisfrenata* (Günther, 1858)	12				
*Lycodonchapaensis* (Angel & Bourret, 1933)	11, 12				
*Lycodondavisonii* (Blanford, 1878)	3, 6				
*Lycodonflavozonatus* (Pope, 1928)	11, 12				
*Lycodonfutsingensis* (Pope, 1928)	1, 6, 11, 12				
*Lycodonmeridionalis* (Bourret, 1935)	6, 11, 12				
*Lycodonchapaensis* (Angel & Bourret, 1933)	9, 12				
*Lycodonruhstrati* (Fischer, 1886)	1, 5, 6, 10, 12				
*Lycodonseptentrionalis* (Günther, 1875)	2				
*Lycodonsubcinctus* Boie, 1827	1, 5, 11, 12				
*Oligodoncatenatus* (Blyth, 1855)	5				
*Oligodonchinensis* (Günther, 1888)	6				
*Oligodoncyclurus* (Cantor, 1839)	11, 12				
*Oligodonfasciolatus* (Günther, 1864)*	12				
*Oligodontaeniatus* (Günther, 1861)	6				
*Oreocryptophisporphyraceus* (Cantor, 1839)	6	VU			
*Pareascarinatus* Wagler, 1830	8, 11, 12				
*Pareashamptoni* (Boulenger, 1905)	3, 6, 8, 11, 12				
*Pareasmacularius* Theobald, 1868*	13				
*Pareasmargaritophorus* (Jan, 1866)	11, 12				
*Pseudoxenodonbambusicola* (Vogt 1922)	5				
*Pseudoxenodonmacrops* (Blyth, 1855)*	13				
*Ptyaskorros* (Schlegel, 1837)	1, 2, 3, 6, 10, 11, 12	EN	NT		
*Ptyasmucosa* (Linnaeus, 1758)	1, 2, 3, 6, 10	EN		IIB	
*Ptyasmulticinctus* (Roux, 1907)	3, 5, 6, 11, 12				
*Rhabdophischrysargos* (Schlegel, 1837)	1, 2, 10				
*Rhabdophishelleri* (Schmidt, 1925)	1, 3, 5, 6, 10, 11, 12				
*Rhabdophisnigrocinctus* (Blyth, 1856)	12				
*Rhabdophissiamensis* (Mell, 1931)	12				
*Sibynophischinensis* (Günther, 1889)	12				
*Trimerodytespercarinatus* Boulenger, 1899	3, 5, 6, 11, 12				
*Trimerodytesaequifasciatus* (Barbour, 1908)	6				
*Xenochrophistrianguligerus* (Boie, 1827)	12				
Pseudaspididae					
*Psammodynastespulverulentus* (Boie, 1827)	1, 3, 6, 8, 10				
Elapidae					
*Bungarusfasciatus* (Schneider, 1801)	1, 2, 3, 6, 10, 11, 12	EN			
*Bungarusmulticinctus* Blyth, 1861	1, 2, 3, 6, 8, 10, 11, 12		VU		
*Bungarusslowinskii* Kuch, Kizirian, Nguyen, Lawson, Donnelly & Mebs, 2005	6		VU		
*Najaatra* Cantor, 1842	1, 2, 5, 6, 10, 12	EN	VU	IIB	
*Ophiophagushannah* (Cantor, 1836)	1, 2, 3, 6, 10	CR	VU	IB	I
*Sinomicrurusmacclellandi* (Reinhardt 1844)	1, 5, 6, 10, 12				
Homalopsidae					
*Myrrophischinensis* (Gray, 1842)	1, 6, 10, 11				
*Hypsiscopusplumbea* (Boie 1827)	1, 3, 5, 6, 10, 12				
Viperidae					
*Trimeresurusalbolabris* Gray, 1842	1, 2, 3, 6, 10, 11, 12				
*Ovophistonkinensis* (Bourret, 1934)	11, 12				
*Protobothropsmucrosquamatus* (Cantor, 1839)	3, 5, 6, 11, 12				
*Trimeresurusstejnegeri* Schmidt, 1925	3, 6, 8				
TESTUDINES					
Platysternidae					
*Platysternonmegacephalum* Gray, 1831	1, 2, 3, 4, 10	EN	CR	IB	I
Testudinidae					
*Indotestudoelongata* (Blyth, 1854)	1, 10	EN	CR	IIB	II
*Manouriaimpressa* (Günther, 1882)	1, 2, 3, 4, 10, 11	VU	EN	IIB	II
Geoemydidae					
*Cuoracyclornata* Blanck, Mccord & Le, 2006	1, 10	CR		IB	II
*Cuoragalbinifrons* (Bourret, 1939)	1, 2, 3, 4, 5, 10, 11	EN	CR	IB	I
*Cuoramouhotii* (Gray 1862)	1, 2, 3, 5, 10, 11		EN	IIB	II
*Cyclemysoldhamii* Gray, 1863	3, 4		EN	IIB	II
*Geoemydaspengleri* (Gmelin, 1789)	3, 4, 10		EN	IIB	II
*Heosemysgrandis* (Gray, 1860)	3, 4	VU	CR	IIB	II
*Malayemyssubtrijuga* (Schlegel & Müller, 1845)	2	VU	NT	IIB	II
*Mauremyssinensis* (Gray, 1834)	1, 10		CR		II
*Sacaliaquadriocellata* (Siebenrock, 1903)	1, 10		CR	IIB	II
Trionychidae					
*Paleasteindachneri* (Siebenrock, 1906)	1, 2, 10, 11	VU	CR	IIB	II
*Pelodiscusvariegatus* Farkas, Ziegler, Pham, Ong & Fritz, 2019	1, 2, 3, 5, 10		VU		
*Rafetusswinhoei* (Gray, 1873)	1	CR	CR	IB	II

## References

[B12222710] Böhm M et al. (2013). The conservation status of the World’s reptiles. Biological Conservation.

[B12222850] Bourret R. (1937). Notes herpetologiques sur l'Indochine francaise.

[B11973371] CITES (2023). Convention on international trade in endangered species of wild fauna and flora. Appendices I, II, and III.

[B11973389] Dang N. T., Tran K., Dang H. H., Nguyen C., Nguyen T. N., Nguyen H. Y., Dang T. D. (2007). Vietnam Red Data Book. Part I. Animals..

[B11973380] Darevsky I. S., NL Orlov, Ho C. T. (2004). Two new lygosomine skinks of the genus *Sphenomorphus* Fitzinger, 1843 (Sauria, Scincidae) from northern Vietnam. Russian Journal of Herpetology.

[B11973465] Dau V. Q., Pham A. H., Bui H. T., Vu H. H.T., Bui T. B. (2019). New records of snakes (Reptilia: Squamata: Serpentes) from Pu Luong Nature Reserve, Thanh Hoa, Province. Proceedings of the 4^th^ National Scientific Conference on Amphibians and Reptiles in Vietnam.

[B12224909] Hammer O., Harper D. A.T., Ryan P. D. (2001). PAST: Paleontological statistical software package for education and data analysis. Paleontologica Eletronica.

[B11973474] Hauser S. (2017). On the validity of *Pareasmacularius* Theobald, 1868 (Squamata: Pareidae) as a species distinct from *Pareasmargaritophorus* (Jan in Bocourt, 1866). Tropical Natural History.

[B12222902] Ha V. N., Dong T. H., Ha V. N., Do Q. T., Ngo A. S., Luu Q. V., Hoang T. L., Bui V. K. (2024). An updated list of snakes from Nam Dong Endangered Gymnosperm Conservation Area, Thanh Hoa Province. Vietnam Journal of Agriculture and Rural development.

[B11973483] Hecht V. L., Pham C. T., Nguyen T. T., Nguyen T. Q., Bonkowski M., Ziegler T. (2013). First report on the herpetofauna of Tay Yen Tu Nature Reserve, northeastern Vietnam. Biodiversity Journal.

[B11973494] Hikida T., Lau M. W.N., Ota H. (2001). A new record of the Vietnamese five-lined skink, *Eumecestamdaoensis* (Reptilia: Scincidae), from Hong Kong, China, with special reference to its sexual dimorphism. Natural History Journal of Chulalongkorn University.

[B11973503] Hoang T. N., Ngo C. D., Hoang Q. X. (2020). Species composition of amphibians and reptiles in the North Central Vietnam. Vietnam Journal of Science and Technology.

[B12223099] Huang Q. (2004). *Pareasmacularius* Theobald, 1868 Should be a Junior Synonym of Pareasmargaritophorus (Jan,1866. Sichuan Journal of Zoology.

[B11973512] IUCN The IUCN Red List of Threatened Species. Version 2024-1. https://www.iucnredlist.org.

[B11973528] Luu V. Q., Ziegler T., Ha N. V., MD Le, Hoang T. T. (2019). A new species of *Lycodon* Boie, 1826 (Serpentes: Colubridae) from Thanh Hoa Province, Vietnam. Zootaxa.

[B11973520] Luu V. Q. (2022). Amphibians and Reptiles of Nam Dong Valuable Gymnosperm Conservation Area.

[B12213577] Marsili L., Casini S., Mori G., Ancora S., Bianchi N., D'Agostino A., Ferraro M., Fossi M. C. (2009). The Italian wall lizard (*Podarcissicula*) as a bioindicator of oil field activity. Science of the Total Environment.

[B12213606] Nguyen Q. T., Le D. M., Pham V. A., Ngo N. H., Hoang V. C., Pham T. C., Ziegler T. (2015). Two new species of *Cyrtodactylus* (Squamata: Gekkonidae) from the karst forest of Hoa Binh Province, Vietnam. Zootaxa.

[B12213619] Nguyen Q. T., Pham V. A., Ziegler T., Ngo T. H., Le D. M. (2017). A new species of *Cyrtodactylus* (Squamata: Gekkonidae) and the first record of C. otai from Son La Province, Vietnam. Zootaxa.

[B11973538] Nguyen S. N., Yang L. X., Le T. N.T., Nguyen L. T., Orlov L. N., Hoang C. V., Nguyen T. Q., Jin Q. J., Rao Q. D., Hoang T. N., Che I., Murphy WR, Zhang P. Y. (2014). DNA barcoding of Vietnamese benttoed geckos (Squamata: Gekkonidae: Cyrtodactylus) and the description of a new species. Zootaxa.

[B11973565] Nguyen S. V, Ho C. T, Nguyen T. Q (2009). Herpetofauna of Vietnam.

[B11973587] Nguyen T. K., Nguyen D. T., Hoang N. T., Truong T. N. (2011). Herpetofauna of Pu Hu Nature Reserve, Thanh Hoa Province. The 4^th^ National Conference on Ecology and Biological Resources,.

[B12222751] Nguyen T. Q., Nguyen T. T., Schmitz A., Orlov N., Ziegler T. (2010). A new species of the genus *Tropidophorus* Duméril & Bibron, 1839. from Vietnam. Zootaxa.

[B11973596] Nguyen T. Q., Schmitz A., Nguyen T. T., Orlov N. L., Böhme W., Ziegler T. (2011). Review of the genus *Sphenomorphus* Fitzinger, 1843 (Squamata: Sauria: Scincidae) in Vietnam, with description of a new species from northern Vietnam and southern China and the first record of *Sphenomorphusmimicus* Taylor, 1962 from Vietnam. Journal of Herpetology.

[B11973624] Nguyen T. T., Nguyen L. T., Pham T. V., Nguyen M. D., Nguyen Q. T, Nguyen S. N. (2015). Preliminary results on species composition of the reptiles in Pu Luong Nature Reserve, Thanh Hoa Province.

[B11973634] Nguyen T. T., Nguyen Q. H., Luu V. Q. (2018). New record of Bent-toed Gecko (*Cyrtodactylusbobrovi*) from Cuc Phuong National Park. Journal of Forestry and Technology.

[B11973607] Nguyen T. V., Pham C. T., Nguyen T. Q. (2016). New records and an updated list of snakes (Squamata: Serpentes) from Xuan Lien Nature Reserve, Thanh Hoa Province, Vietnam. Journal of Biology.

[B11973652] Pham A. V., Le D. T., Nguyen L. S.H., Ziegler T., Nguyen T. Q. (2015). New provincial records of skinks (Squamata: Scincidae) from northwestern Vietnam. Biodiversity Data Journal.

[B11973671] Pham A. V., Ziegler T., Nguyen T. Q. (2020). New records and an updated list of snakes from Son La Province. Vietnam. Biodiversity Data Journal.

[B11973694] Pham T. C., Hoang V. C., Nguyen T. Q., Chu T. T., Nguyen T. T. (2012). Species composition of the herpetofauna of Xuan Lien Nature Reserve, Thanh Hoa Province. Proceedings of the second National Workshop on Herpetology of Vietnam, Vinh University Publishing House.

[B11973662] Pham V. A., Nguyen L. H.S., Nguyen Q. T. (2014). New records of snakes (Squamata: Serpentes) from Son La Province, Vietnam. Herpetology Notes.

[B12213631] Pham V. A., Le D. M., Ngo T. H., Ziegler T., Nguyen Q. T. (2019). A new species of *Cyrtodactylus* (Squamata: Gekkonidae) from northwestern Vietnam. Zootaxa.

[B12213642] Pham V. A., Pham T. C., Le D. M., Ngo T. H., Ong V. A., Ziegler T., Nguyen Q. T. (2023). *Achalinusquangi*, a new odd-scaled snake species from Vietnam. Zootaxa.

[B12222719] Rowley J., Brown R., Bain R., Kusrini M., Inger R., Stuart B., Wogan G., Thy N., Chan-ard T., Cao T. T., Diesmos A., Iskandar D. T., Lau M., Ming L. T., Makchai S., Nguyen T. Q., Phimmachak S. (2010). Impending conservation crisis for southeast Asian amphibians. Biology Letters.

[B12222858] Schulz K. D. (2010). Synopsis of the Variation in the *Orthriophistaeniurus* Subspecies Complex, with Notes to the Status of Coluber taeniurus pallidus Rendahl, 1937 and the Description of a new Subspecies (Reptilia: Squamata: Serpentes: Colubridae. Sauria.

[B11973704] Simmons J. E. (2002). Herpetological collecting and collections management. Revised edition. Society for the Study of Amphibians and Reptiles.. Herpetological Circular.

[B12222783] Smith M. A. (1935). The fauna of British India, including Ceylon and Burma. Reptilia and Amphibia.

[B11973713] Smith M. A. (1943). The fauna of British India, Ceylon and Burma, including the whole of the Indo-Chinese Subregion. Reptilia and Amphibia. Vol. III. Serpentes. Taylor and Francis (London).

[B11973722] Suntrarachun S., Chanhome L., Hauser S., Sumontha M., Kanya K. (2020). Molecular phylogenetic support to the resurrection of *Pareasmacularius* from the synonymy of *Pareasmargaritophorus* (Squamata: Pareidae. Tropical Natural History.

[B11973732] Taylor E. H. (1965). The serpents of Thailand and adjacent waters. Kansas University Science Bulletin.

[B11973751] Vietnam The Government of (2021). The Governmental Decree No. 84/2021/NĐ-CP, dated on 22th September 2021, on management of endangered, precious, and rare species of wild plants and animals.

[B11973759] Province The People's Committee of Thanh Hoa Thanh Hoa Province Portal. http://thanhhoa.gov.vn/gioi-thieu.

[B11973787] Uetz P., Freed P., Aguilar R., Reyes F., Kudera J., Hošek J. http://www.reptile-database.org. The Reptile Database.

[B12223039] Wang K., Yu Z. B., Vogel G., Che J. (2020). Contribution to the taxonomy of the genus Lycodon H. Boie in Fitzinger, 1827 (Reptilia: Squamata: Colubridae) in China, with description of two new species and resurrection and elevation of *Dinodonseptentrionalechapaense* Angel, Bourret, 1933. Zoological Research.

[B11973797] Wang P., Che J., Liu Q., Li K., Jin J. Q., Jiang K., Shi L., Guo P. (2020). A revised taxonomy of Asian snail-eating snakes *Pareas* (Squamata, Pareidae): evidence from morphological comparison and molecular phylogeny. ZooKeys.

[B11973810] Zaher H., Murphy R. W., Arredondo J. C., Graboski R., Machado-Filho P. R., Mahlow K. (2019). Large-scale molecular phylogeny, morphology, divergence-time estimation, and the fossil record of advanced caenophidian snakes (Squamata: Serpentes). PLoS ONE.

[B12222741] Ziegler T., Nguyen T. Q., Schmitz A., Stenke R., Rösler H. (2008). A new species of *Goniurosaurus* from Cat Ba Island, Hai Phong, northern Vietnam (Squamata: Eublepharidae). Zootaxa.

[B11973822] Ziegler T., Rauhaus A., Tran T. D., Pham C. T., Van Schingen M., Dang P. H., Le D. M., Nguyen T. Q. (2015). Die Amphibien- und Reptilienfauna der Me-Linh-Biodiversitätsstation in Nordvietnam. Sauria.

